# Supplementary material for: Modeling glioblastoma heterogeneity as a dynamic network of cell states
Source: Mol Syst Biol. 2021 Sep 16;17(9):e10105. doi: 10.15252/msb.202010105 (PMC8444284; doi:10.15252/msb.202010105)
Supplement: Supplementary file 6 — Source Data for Figure 5 [file MSB-17-e10105-s004.zip › Figure5A_sourcedata/GSEA_3017/nosigdb_stateC.GseaPreranked.1622795002785/SUVA_AC_PMID_31327527.html]

Details for gene set SUVA\_AC\_PMID\_31327527[GSEA]

|  || Dataset | state13017 |
| Phenotype | NoPhenotypeAvailable |
| Upregulated in class | na\_neg |
| GeneSet | SUVA\_AC\_PMID\_31327527 |
| Enrichment Score (ES) | -0.66866076 |
| Normalized Enrichment Score (NES) | -3.063508 |
| Nominal p-value | 0.0 |
| FDR q-value | 0.0 |
| FWER p-Value | 0.0 |
Table: GSEA Results Summary

  

Fig 1: Enrichment plot: SUVA\_AC\_PMID\_31327527      
 Profile of the Running ES Score & Positions of GeneSet Members on the Rank Ordered List

  

| PROBE | GENE SYMBOL | GENE\_TITLE | RANK IN GENE LIST | RANK METRIC SCORE | RUNNING ES | CORE ENRICHMENT || 1 | GATM |  |  | 272 | -0.270 | -0.4521 | No |
| 2 | SLC1A3 |  |  | 307 | -0.281 | -0.4880 | No |
| 3 | PLTP |  |  | 397 | -0.319 | -0.6166 | No |
| 4 | PMP2 |  |  | 424 | -0.337 | -0.6338 | No |
| 5 | RAB31 |  |  | 445 | -0.349 | -0.6395 | Yes |
| 6 | BCAN |  |  | 449 | -0.351 | -0.6153 | Yes |
| 7 | EDNRB |  |  | 462 | -0.367 | -0.6056 | Yes |
| 8 | FABP7 |  |  | 495 | -0.411 | -0.6270 | Yes |
| 9 | AGT |  |  | 497 | -0.412 | -0.5942 | Yes |
| 10 | F3 |  |  | 505 | -0.432 | -0.5702 | Yes |
| 11 | DBI |  |  | 514 | -0.440 | -0.5473 | Yes |
| 12 | CST3 |  |  | 520 | -0.452 | -0.5182 | Yes |
| 13 | TTYH1 |  |  | 540 | -0.496 | -0.5098 | Yes |
| 14 | HOPX |  |  | 546 | -0.513 | -0.4756 | Yes |
| 15 | RAMP1 |  |  | 548 | -0.517 | -0.4341 | Yes |
| 16 | SPARCL1 |  |  | 557 | -0.544 | -0.4025 | Yes |
| 17 | S100B |  |  | 561 | -0.561 | -0.3608 | Yes |
| 18 | SPARC |  |  | 567 | -0.575 | -0.3214 | Yes |
| 19 | S100A16 |  |  | 569 | -0.582 | -0.2744 | Yes |
| 20 | GFAP |  |  | 574 | -0.659 | -0.2262 | Yes |
| 21 | CLU |  |  | 581 | -0.690 | -0.1789 | Yes |
| 22 | ATP1B2 |  |  | 585 | -0.714 | -0.1244 | Yes |
| 23 | PMP22 |  |  | 590 | -0.837 | -0.0613 | Yes |
| 24 | MT3 |  |  | 591 | -0.837 | 0.0087 | Yes |
Table: GSEA details [plain text format]

  

Fig 2: SUVA\_AC\_PMID\_31327527: Random ES distribution      
 Gene set null distribution of ES for **SUVA\_AC\_PMID\_31327527**
